# Dependence of the Ripeness Stage on the Antioxidant and Antimicrobial Properties of Walnut (*Juglans regia* L.) Green Husk Extracts from Industrial By-Products

**DOI:** 10.3390/molecules26102878

**Published:** 2021-05-13

**Authors:** Daniela Soto-Madrid, Marlen Gutiérrez-Cutiño, Josué Pozo-Martínez, María Carolina Zúñiga-López, Claudio Olea-Azar, Silvia Matiacevich

**Affiliations:** 1 Food Properties Research Group (INPROAL), Department of Food Science and Technology, Technological Faculty, Universidad de Santiago de Chile (USACH), Obispo Umaña 050, Estación Central, Santiago 9170201, Chile; daniela.sotom@usach.cl; 2 Molecular Magnetism & Molecular Materials Laboratory (LM4), Department of Chemistry of Materials, Chemistry and Biology Faculty, Universidad de Santiago de Chile (USACH), Av. Lib. Bernardo O’Higgins 3363, Estación Central, Santiago 9170022, Chile; marlen.gutierrez@usach.cl; 3 Center for the Development of Nanoscience and Nanotechnology (CEDENNA), Estación Central, Santiago 9170022, Chile; 4 Department of Inorganic and Analytical Chemistry, Universidad de Chile, Sergio Livingstone 1007, Independencia, Santiago 8380492, Chile; jspozom@yahoo.es (J.P.-M.); mczuniga@ciq.uchile.cl (M.C.Z.-L.); colea@uchile.cl (C.O.-A.)

**Keywords:** walnut green husk, ripeness stage, antioxidant activity, antimicrobial activity, *Escherichia coli*

## Abstract

Walnut green husk (WGH) is a waste generated by the walnut (*Juglans regia L.*) harvest industry. It represents a natural source of polyphenols, compounds with antioxidant and antimicrobial activities, but their activity could be dependent on the ripeness stage of the raw material. In this study, the effect of the different ripeness stages—open (OH) and closed (CH) husks—on the antioxidant and antimicrobial properties of WGH extracts were analyzed, emphasizing the influence of the extracts in inhibiting *Escherichia coli* growth. The ripeness stage of WGH significantly affected the antioxidant activity of the extracts. This was attributed to the different polyphenol profiles related to the mechanical stress when the husk opened compared to the closed sample. The antimicrobial activity showed inhibition of *E. coli* growth. OH-extracts at 96 µg/mL caused the lowest specific growth rate (*µ*_max_ = 0.003 h^−1^) and the greatest inhibition percentage (*I* = 93%) compared to CH-extract (*µ*_max_ = 0.01 h^−1^; *I* = 69%). The obtained results showed the potential of the walnut green husk, principally open husk, as an economical source of antioxidant and antimicrobial agents with potential use in the food industry.

## 1. Introduction

The walnut industry has had a steady growth worldwide, in terms of the volumes traded and the surface area dedicated to this crop. Chile ranks second among the leading exporters of walnuts worldwide [1]. However, this industry generates a large amount of waste that mainly corresponds to the walnut green husk (exocarp and mesocarp), equivalent to approximately 20% of the total walnut production [2]. Walnut green husk is a waste produced during the harvest season and currently has few uses. One of them is as a fertilizer because it is a tissue rich in organic matter. However, it is also used as a coloring for woods or a replacement for Chinese ink [3].

Several studies have shown that walnut green husk is rich in polyphenolic compounds with an antimicrobial and antioxidant capacity [3,4,5]. Therefore, walnut green husks represent a natural source of active compounds (polyphenols) and are ideal for extracting them. However, to obtain active compounds, extraction is a critical step due to process variability and complexity in terms of solubility, chemical structure, the relative quantity of the compound in each sample, and no standardized extraction protocol [6]. The extraction of these compounds can be carried out through conventional solid–liquid extraction, which involves heating, boiling, or stirring at room temperature. However, hydrolysis and oxidation of the compounds during extraction and a low yield of the active compounds in the extract are disadvantages of these processes [7]. In recent years, different extraction methods and process conditions have been used for polyphenol extraction from the walnut green husk [3,8,9,10,11,12]. The most common is ultrasound-assisted extraction (UAE), using solvents such as water, ethanol, and methanol [10].

UAE has gained importance because it is a simple, efficient, and sustainable procedure. It allows better cellular penetration of solvents, shorter extraction times, and a higher extraction performance of phenolic compounds from a plant matrix, even at lower temperatures than other extraction methods [13]. This method creates hotspots, which can dramatically improve solvent penetration into cell components, and even induce cell breakdown, thus provoking the release of the inner cell content [7]. Therefore, optimizing extraction processes by UAE is essential.

Furthermore, phenolic compounds are secondary metabolites, which occur in abundance in plant materials. They belong to a large and heterogeneous group of biologically active compounds, involving physiological processes on fruit growth and affecting different aspects of pre-and post-harvest fruit life [14]. Their synthesis depends on numerous enzymes involved in different metabolic pathways [15]. Their metabolism is wholly integrated into morphological and biochemical regulatory patterns of plants [16]. The content of phenolic compounds depends on many environmental conditions, such as geographical location and climatic conditions, and the cultivar’s genotype [16]. Specifically, it depends on the nut’s developmental stage, where a negative correlation was observed between growth and the concentration of the phenolic compounds [16]. Therefore, it is essential to consider the ripeness stage for the extraction of phenolic compounds from the walnut green husks, due to it having seldom been studied in the literature [3,8,10,11,12].

Moreover, walnut green husks extracts showed antimicrobial properties against pathogenic bacteria such as *B*. *cereus*, *B. subtilis*, *S. aureus*, *E. coli,* and *P. aeruginosa* [3], where Gram-positive (+) bacteria were the most sensitive to being inhibited by the extracts. However, this antimicrobial activity was only performed on solid medium methodology by contact. Hence, the study of its influences on bacterial growth kinetics could be helpful for liquid food applications.

Accordingly, the present study aimed to evaluate the effect of the ripeness stage on the antioxidant and antimicrobial properties of walnut green husks extracts, emphasizing the influence of the extracts on *Escherichia coli* growth kinetic parameters. The industrial importance of this study is related to obtaining information about the valorization and use of these walnut wastes that have contemporary limited use.

## 2. Results and Discussion

### 2.1. Characterization of Walnut Green Husks

#### 2.1.1. Proximal Analysis

**Figure 1:**
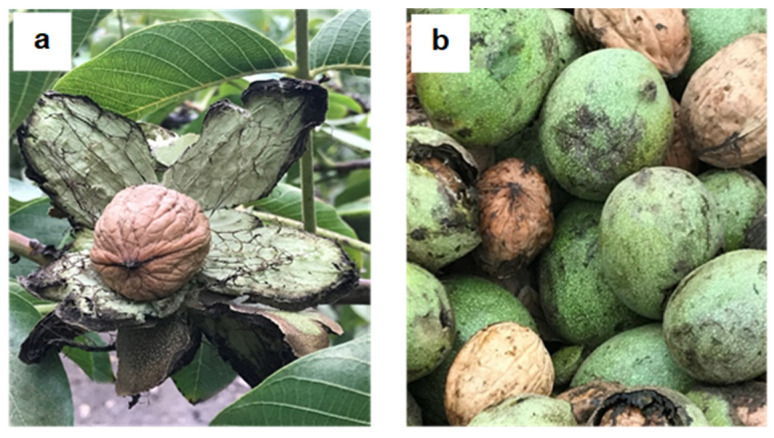
Samples of walnut green husks with different ripeness stages: (**a**) ripe walnut green husk with an open husk (OH); and (**b**) unripe walnut green husk with a closed husk (CH).

Ripeness involves biochemical, physiological, and structural changes, modifying the nutritional and fruit proximal composition [17]. However, it was not evident (Table 1), because no significant differences (*p* ≥ 0.05) were observed in the proximal composition between the two samples, despite the different ripeness stages. The biochemical changes during walnut green husk ripening are probably not enough to modify protein, carbohydrate, lipid, and ash levels, as occurs in other vegetable matrices [18]. On the other hand, significant differences (*p* < 0.05) were observed for moisture content, where the open samples (OH) had the lowest content (Table 1). This dehydration was attributed to the highest exposure of the mesocarp to the environmental conditions while it was in the tree because the husk is opening (Figure 1a) [19].

#### 2.1.2. Antioxidant Capacity

Studies in the literature had previously reported that walnut green husks showed antioxidant capacity, which was attributed to the presence of polyphenolic compounds [3,4,5]. Nevertheless, this property has been scarcely studied comparing different ripeness stages and/or among fruits/vegetables of habitual consumption. This comparison is necessary to evaluate the technological viability and value-added of walnut green husk. Therefore, antioxidant capacity was compared between ripeness stages (OH and CH) and with fruits of known antioxidant activity (INTA database), using the ORAC methodology [20].

The obtained results showed the higher antioxidant capacity of the OH sample (47.94 µmol Trolox/g sample dry weight). This value was similar to the antioxidant capacities of dried fig (45.7 µmol Trolox/g sample dry weight), princess grape (43.12 µmol Trolox/g sample dry weight), and fresh coconut (39.48 µmol Trolox/g sample dry weight). In comparison, the antioxidant capacity of the CH sample (34.97 µmol Trolox/g sample dry weight) was comparable to an edranol avocado (36.55 µmol Trolox/g sample dry weight) and roasted peanuts (28.43 µmol Trolox/g sample dry weight) [21]. These results are in agreement with the work of Laroze et al. [22]. They indicated that the external parts of fruits, vegetables, and other lignocellulosic parts such as skins, husks, and seeds have a higher content of antioxidant phenolic compounds than the corresponding internal fractions (pulp of fruits). The difference in antioxidant activity between the ripeness stages could be attributed to different phenolic compound profiles (Table 2).

These results indicate that these by-products, which are currently industrial waste, have antioxidant activity comparable to regular products. Therefore, it is of interest to use the polyphenols extracted from walnut green husk for a technological purpose based on the current trend of a circular economy.

#### 2.1.3. Identification of Phenolic Compound Profiles

The control extraction methodology extracted polyphenolic compounds (point 3.4), were identified by reversed-phase high-performance liquid chromatography (HPLC-RP). The polyphenol presence was compared to 12 synthetic polyphenol standards previously found by other researchers in walnut green husk [23,24,25] (Table 2). Nine polyphenols were identified in the OH sample and seven were observed in the CH sample (Table 2). However, polyphenol profile differences were observed, comparable to those found in the literature, probably due to different solvents used in the extractions. Therefore, solvent and extraction conditions are critical parameters to extract other polyphenols not previously identified, because compounds could have different polarities.

Gallic acid was the polyphenol identified with the highest intensity on both samples (data not shown). Nevertheless, a greater area (4978 mUA/min) was observed for the OH sample compared to the CH sample (3585 mUA/min); this could be related to a higher concentration of this polyphenol in that sample. The higher antioxidant capacity of the OH sample may be related to the higher concentration of this phenolic acid, which several authors have previously described in the walnut green husk [23,24,25].

On the other hand, differences in the composition of extracts were observed because the OH sample presented caffeic acid and polydatin, unlike CH, which could be related to the observed antioxidant capacity differences [26,27]. Caffeic acid is a hydroxycinnamic acid found in plants as an intermediate for lignin (cell wall polymer) biosynthesis. Lignification of plant tissue occurs in response to various environmental factors such as mechanical stress [28], which could occur while a green husk is opened. Meanwhile, polydatin is a stilbene polyphenol, a precursor of resveratrol, and a natural phenol produced in the response of mechanical lesion [29], also attributed to the husk opening (OH).

Therefore, the differences previously observed for antioxidant activity due to the ripeness stage were attributed to different polyphenol profiles due to the presence of more identified antioxidant polyphenols on the OH sample, related to the mechanical stress of husk opening and the highest peak area of gallic acid polyphenol.

It is also essential to consider that polyphenols detected on ripped walnut green husks (OH sample) showed antioxidant properties that may benefit human health [30,31] (Ramassamy, 2006; Vita, 2005). These antioxidant polyphenols were gallic acid, caffeic acid, protocatechuic acid, catechin, ferulic acid, quercetin, and polydatin. It indicates that walnut green husk could be a new source to obtain antioxidant polyphenols. Considering their benefits to human health and high instability and degradation [6], their protection is essential during extraction and storage.

Results revealed that the OH sample showed the highest antioxidant capacity. Therefore, it was selected for optimization through ultrasound-assisted extraction.

### 2.2. Optimization of the Extraction Process of the Walnut Green Husk

Considering the different process extractions was used in the literature for walnut green husk, there is no standardized extraction process for this the sub-product [3,8,9,10,11]. In this study, a central composite design was used to optimize the extraction process for this raw material to obtain the maximum antioxidant capacity of the extract (Table 3).

**Figure 2:**
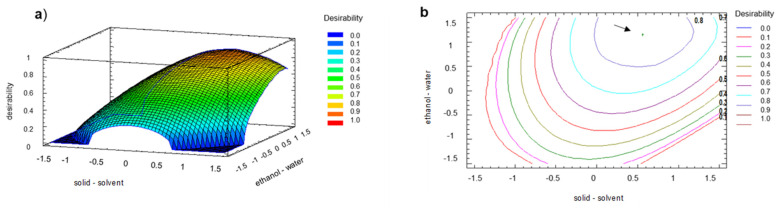
Results of extraction optimization by (**a**) desirability function and (**b**) contour plots with optimum condition.

The responses studied using the design were the total polyphenolic content (TPC) and antioxidant capacity measured by ORAC, DPPH, and FRAP. These responses were maximized through a desirability function. The desirability function considered all the studied parameters to obtain an optimal condition that maximized its values, i.e., the highest amount of total polyphenol and the most significant antioxidant capacity. Figure 2a shows the optimal condition for the studied levels, indicating an optimum value of desirability of 0.87 (Figure 2b), which corresponds to an optimal value of the solid/solvent factor of 1:25 (*w/v*) and an optimal value of ethanol/water solvent ratio of 75:25 (*v/v*).

The design generated a nonlinear, quadratic model represented by the following equation:

(1) $$y=a_{0}+a_{1}x_{1}+a_{2}x_{2}-a_{3}x_{1}^{2}+a_{4}x_{1}x_{2}-a_{5}x_{2}^{2}$$

The response (*y*) corresponds to the dependent variables associated with each factor/level combination, *a*_0_ to *a*_5_ are the regression coefficients of the respective variables, and *x*_1_, *x*_2_ corresponds to the two independent variables. The terms *x*_1_*x*_2_ and *x*_i_^2^ represent the interaction and the quadratic terms, respectively.

The predicted response values from the desirability function were 100 mg GAE/g sample dry weight (TPC), 191 mg Trolox/g sample dry weight (DPPH), 123 mg FeSO_4_/g sample dry weight (FRAP), and 158 µmol Trolox/g sample dry weight (ORAC).

To validate the model, three independent runs at the determined optimized conditions were performed and compared to the predicted values. Results showed that although higher values (approximately 10%) of antioxidant capacities were observed, no significant differences (*p* < 0.05) were obtained between predicted and performed values. The coefficient of variation (CV) was lower than 10% and the coefficient of determination (R^2^) between the experimental values of each evaluated factor and the predicted values suggested by the desirability function was higher than 0.95, validating the model. It is important to note that the values of polyphenol content and antioxidant capacity obtained using the optimized conditions from this study were higher than those values described previously in the literature [3,10,11]. Therefore, using a desirability function maximizes several parameters, allowing them to obtain satisfactorily and validated optimized conditions for the extraction process using walnut green husk as the polyphenol source.

The optimized extracts showed different phenolic compositions from that obtained by the control extraction. It was attributed to the solvent polarity used because the hydroethanolic solvent used in this study was more polar than the standard extraction solvent (acetone–water–acetic acid). Therefore, apolar polyphenols were identified when standard extraction [32] was used, as compared to the optimized extractions of this study.

### 2.3. Evaluation of the Antioxidant and Antimicrobial Capacity of the Optimized Extracts of Walnut Green Husk

#### 2.3.1. Antioxidant Capacity

**Figure 3:**
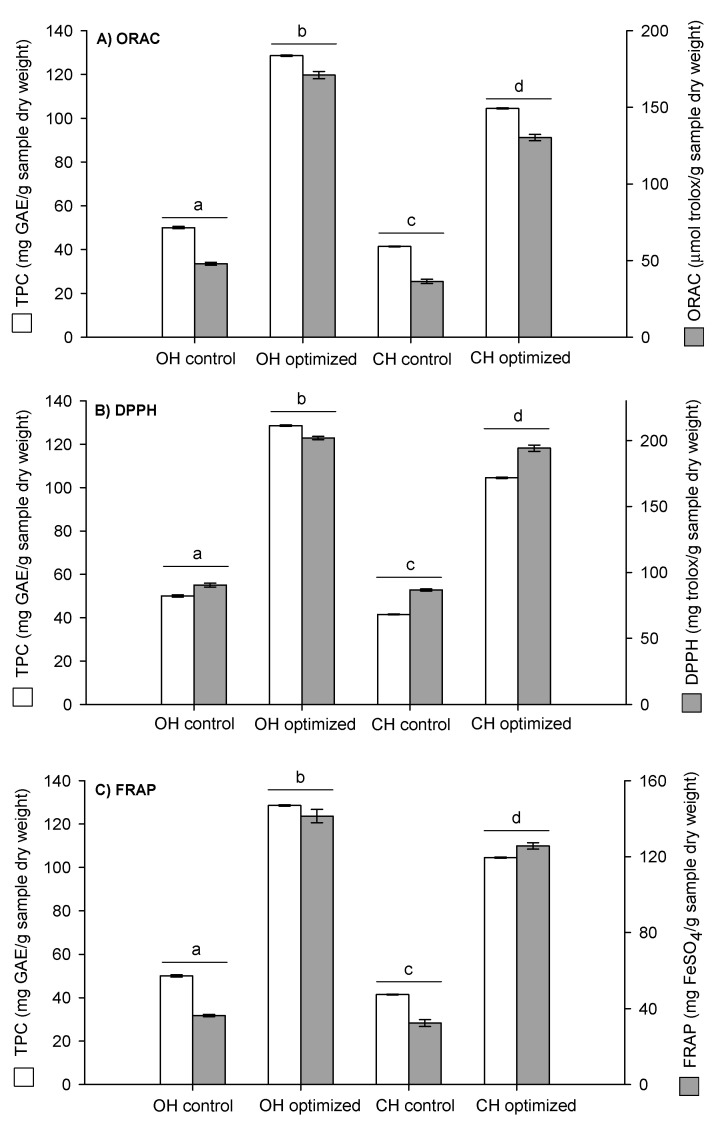
Total polyphenol content (TPC) and antioxidant activity of the extracts of the walnut green husks (OH: open husk and CH: closed husk) measured by (**a**) ORAC: oxygen radical absorbance capacity; (**b**) DPPH radical scavenging activity; and (**c**) FRAP: ferric reducing antioxidant power. Different letters (a, b, c, d) indicate significant differences *(p* < 0.05) between samples.

In this study, the antioxidant capacities of the optimized extracts samples (OH and CH) were measured through a test based on hydrogen atom transfer (HAT) as ORAC methodology, and through a test based on electron transfer (ET) as DPPH and FRAP methods. Antioxidant activity and total polyphenol content were compared between the control and the optimized extraction of the two samples (OH and CH) (Figure 3).

The obtained results indicated that the highest antioxidant capacity of the optimized samples corresponded to the OH sample (DPPH: 202 ± 1.2 mg Trolox/g sample dry weight, FRAP: 141 ± 3.5 mg FeSO_4_/g sample dry weight, and ORAC: 171 ± 2.4 µmol Trolox/g sample dry weight). These results are attributed to the highest amount of total polyphenol content (TPC) extracted (OH: 129 ± 0.5 mg GAE/g sample dry weight compared to CH: 105 ± 2.3 mg GAE/g sample dry weight). Furthermore, these values confirm the previous characterization results, where the ripeness stage of the walnut green husk was directly related to its antioxidant capacity.

Optimized and control extractions were compared for the two analyzed samples (OH and CH). Figure 3 shows that optimized extracts containing a TPC increased 2.5-fold for both samples. The increase in the antioxidant capacity was 3.6-, 3.9-, and 2.2-fold for ORAC, DPPH, and FRAP, respectively, compared to the control extraction. Therefore, these results confirm the increase in these responses by the optimization design performed.

Therefore, optimized walnut green husk extracts had a higher antioxidant capacity than the control extraction, and could thus be used for technological purposes such as a potential natural additive in replacing synthetic additives.

#### 2.3.2. Antimicrobial Capacity

Walnut green husk extracts showed the highest antimicrobial activity against Gram (+) bacteria [3,10], but the effect of extracts on the bacteria growth kinetic parameters on Gram (−) bacteria has seldom been studied. Therefore, the effect of the concentration of the walnut green husk optimized extracts (OH and CH) on the kinetic growth parameters was studied on *Escherichia coli*, a reference Gram (−) bacterium. Additionally, considering the potential application as active additives, *E. coli* was selected because it is the principal pathogenic bacteria on foods, transmitted through the consumption of contaminated fresh food, due to hygienic deficiencies in processing, and causes intestinal diseases in humans [33].

**Figure 4:**
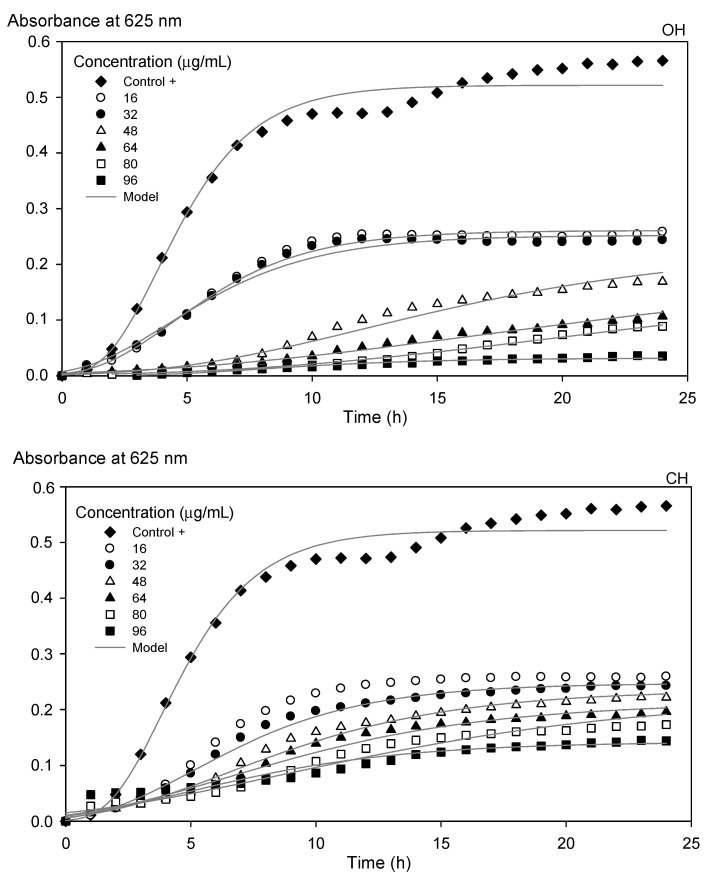
Microbial growth kinetics of *Escherichia coli* under the influence of optimized open husk (OH) and closed husk (CH) extracts. The lines represent the modified Gompertz model.

Kinetic growth parameters were affected by the increasing concentration of the optimized extracts (OH and CH), reflected in the decrease in the specific growth rate (*µ*_max_), obtained by Equation (2), and the increase in the inhibition percentage (*I*%), calculated using Equation (3) (Table 4). OH-extracts at 96 µg/mL showed the lowest *µ*_max_ and the greatest I% (*µ*_max_ = 0.003 h^−1^ and *I* = 93%) compared to CH extract (*µ*_max_ = 0.01 h^−1^ and *I* = 69%). Therefore, OH-extracts showed the greatest antibacterial activity (Figure 4), which can be attributed to the significant differences (*p* < 0.05) in TPC between both samples (OH = 128.6 ± 0.3 mg GAE/g sample dry weight; CH = 104.52 ± 2.3 mg GAE/g sample dry weight). This effect follows previous results because phenolic compounds have been demonstrated to exhibit antibacterial activity [34,35,36,37].

The polyphenol compounds found in both extracts, such as gallic acid, protocatechuic acid, ferulic acid, and juglone (Table 2), have previously been reported to have antimicrobial activity against *E. coli* [23,38,39]. It is also essential to consider that the juglone area obtained by HPLC-RP was higher in the OH sample (32.8 mUA/min) than in the CH sample (27.4 mUA/min). It was determined that this polyphenol is involved in the walnut pathogenic defense mechanism; when the green husk is open, it is more prone to pathogen attack [40,41]. Therefore, polyphenol’s presence on both extracts inhibits the growth of *E. coli* by this antimicrobial activity.

Although Fernandez-Agulló et al. [10] indicated that the walnut green husk did not inhibit the growth of *E. coli* by agar diffusion, in this work, the antimicrobial effect of walnut green husk extracts against this Gram (−) bacterium was demonstrated. It was attributed to the different walnut ripeness stages, variety, and extraction process conditions.

## 3. Materials and Methods

### 3.1. Samples

Walnut green husks were obtained from a walnut tree cultivation (*Juglans regia* L.), Chandler variety, in April 2019 in Cuncumen, Province of San Antonio, V Region, Chile. The orchard has a planting density of 6 × 3 m^2^. Mature trees were used in full production (6 years) to ensure homogeneity. Random sampling was carried out (10 kg) from the center of 10 rows, avoiding the tree tips because they are the most exposed to the environment and pests. Two types of samples were collected: (i) ripe walnut green husk (open husk (OH)): removed manually from the walnut (open stage); and (ii) unripe walnut green husk (closed husk (CH)): removed mechanically by a vibration machine (closed stage) (Figure 1).

### 3.2. Walnut Green Husk Drying

The collected walnut green husks were dried in a forced-air oven (ZenithLab, DHG-9053 A, Jiangsu, China) at 40 °C for 48 h until a constant weight was obtained. Then, dried husks were crushed and ground in Thermomix equipment (Vorwerk, Wuppertal, Germany) and stored at room temperature in glass bottles covered with aluminum foil to protect samples from light.

### 3.3. Proximal Characterization

Proximal analysis was performed on the two representative samples of dried walnut green husks: OH and CH. Moisture, protein, lipid, ash, crude fiber, and non-nitrogen extract contents were determined according to the Official Association of Analytical Chemistry methodologies.

### 3.4. Control Extraction

A control extraction was performed to compare the antioxidant capacity of the walnut green husks with the fruits and vegetables database of the Institute of Nutrition and Food Technology (INTA) of the Universidad de Chile. Extraction with a solvent mix of acetone/water/acetic acid (Merck, Darmstadt, Germany) (70/29.5/0.5% *v/v*) was performed in an ultrasound bath (Daihan, Power sonic 405, Kyonggi-Do, Korea) at 44 kHz frequency for 90 min. Subsequent maceration for 72 h at room temperature, using a solid-to-liquid ratio of 1:25 (g/mL), was developed according to the methodology described by Wu et al. [20]. The extraction was carried out in 1.5 mL amber microtubes, and the temperature in the ultrasound bath was regulated at 25 °C. Finally, the extracts were centrifuged (Hermle, Z306, Wehingen, Germany) under refrigeration conditions (0–5 °C), for 5 min at 7000 rpm, and the liquid phase was collected and stored at 5 °C until later analysis.

### 3.5. Ultrasound-Assisted Extractions

Ultrasound-assisted extractions (UAEs) (Sonics Materials, VCX 500, Newtown, CT, USA) were used to obtain the extracts of the walnut green husks (4 g) by using a high-intensity 3/8-inch tip probe (600 W, 20 kHz, 25 °C, 40 min) and ethanol (Merck, Darmstadt, Germany) or an ethanol–water mixture (75:25) as the solvent. UAE was carried out in a 500 mL beaker immersed in a cold-water bath to control the temperature. The extracts were vacuum-filtered using Whatman N 1 filter paper, and then the solvent was evaporated under low-pressure conditions in a rotary evaporator (Buchi R-100, Flawil, Switzerland) at 40 °C.

### 3.6. Identification of Phenolic Compounds by HPLC-DAD-FLD

Reverse-phase high-performance liquid chromatography (HPLC-RP), coupled to a diode array detector (DAD) and a fluorescence detector (FLD) (Agilent Technologies 1200, Waldbronn, Germany), was used to identify the polyphenolic compounds in extracts (1 g/mL) from walnut green husks. The column used was a C18e Chromolith^®^ HighResolution (150 × 4.60 mm; 5.0 µm) (Merck, Darmstadt, Germany). The mobile phase was a binary mixture of solvents: mobile phase A corresponded to acetonitrile (Merck, Darmstadt, Germany), and mobile phase B corresponded to an acetic acid solution (Merck, Darmstadt, Germany) (2% *v/v*). The identification of phenolic compounds was performed by comparing twelve polyphenol standards (5 mg/L): gallic acid, protocatechuic acid, catechin, caffeic acid, ferulic acid, polydatin, hesperetin, resveratrol, quercetin, myricetin, kaempferol, and hesperidin (Sigma-Aldrich, St. Louis, MO, USA). 

### 3.7. Determination of Total Phenolic Content

A colorimetric assay estimated the total phenolic content (TPC) in the extracts based on procedures described by Singleton et al. [42] with some modifications. Briefly, 0.1 mL of extract of known concentration was added to a 10 mL volumetric flask with 4.9 mL of distilled water and 0.5 mL of Folin–Ciocalteu reagent (Merck, Darmstadt, Germany), followed by 1.7 mL of Na_2_CO_3_ (20% *w/v*, Merck, Darmstadt, Germany) addition. Then, distilled water was added until it reached 10 mL. The reactive mixture was allowed to stand for 2 h in darkness. As an indicator of TPC, the formation of blue color was quantified at 740 nm using a UV–vis spectrophotometer (Shimadzu UVmini-1240, Kyoto, Japan). Gallic acid (Merck, Darmstadt, Germany) was used for constructing the standard curve (2.5 to 125 µg/mL). Results were expressed as milligrams of gallic acid equivalents/g sample dry weight (mgGA/g dw). All assays were performed in triplicate.

### 3.8. Antioxidant Activity

Extracts were diluted to obtain a lineal concentration on the standard curve, being used a different dilution for each extract, depending on the experimental design.

#### 3.8.1. DPPH Radical Scavenging Assay

The effect of scavenging 2,2-diphenyl-1picrylhydrazyl (DPPH) was determined according to the method reported by Brand-Williams et al. [43]. Diluted known concentrations (50 µL) were mixed with 2.75 mL of a methanolic solution containing the DPPH radical (Sigma-Aldrich, St. Louis, MO, USA) (1 mM). The mixture was stirred and left in the dark for 30 min, and subsequently its absorbance at 517 nm was measured using a UV–vis spectrophotometer (Shimadzu UVmini-1240, Kyoto, Japan). The standard curve was constructed using Trolox (Sigma-Aldrich, St. Louis, MO, USA) (0 to 800 µM), and the results were expressed as mg Trolox/g sample dry weight. All assays were performed in triplicate.

#### 3.8.2. Ferric Reducing Antioxidant Power (FRAP)

FRAP assay was performed according to Benzie and Strain [44]. FRAP is based on the ability to reduce yellow ferric tripyridyltriazine complex (Fe (III)-TPTZ) to blue ferrous complex (Fe (II)-TPTZ) by electron-donating phenolic compounds in an acidic medium, which is measured as an absorbance change of ferrous TPTZ complex. The FRAP reagent was prepared by mixing acetate buffer (300 mM, pH 3.6), TPTZ solution (10 mM TPTZ in 40 mM HCl), and FeCl_3_·6H_2_O (20 mM in water solution) (Sigma-Aldrich, St. Louis, MO, USA) in a 10:1:1 (*v/v*) ratio. For a 25 µL volume of the known concentration extract, 175 µL FRAP reagent was added to microplate wells, and the mixture was allowed to stand in the dark for 30 min at 37 °C. The absorbance of the samples was measured at a wavelength of 595 nm and compared to a blank, using a microplate reader (Thermo Fisher, Multiskan Go, Vantaa, Finland). A calibration curve was performed using standard ferrous sulfate solutions (0.5 to 1.4 mmol/L). All measurements were made in triplicate, and the results were expressed in mg FeSO_4_/g sample dry weight.

#### 3.8.3. Oxygen Radical Absorbance Capacity (ORAC)

This methodology determines the level of protection afforded by the antioxidant from the extract of the walnut green husks against oxidative damage caused by radicals generated by the aerobic thermal decomposition of AAPH (2,2′-azo-bis(2-amidinopropane) dihydrochloride) (Sigma-Aldrich, St. Louis, MO, USA), using fluorescein as the probe molecule [45]. Fluorescence intensities were measured at 485/528 nm of excitation/emission wavelength (96-well white polystyrene microplate) for one-minute intervals in a microplate-reader (Bio-Teck Instruments, Synergy HT, Winooski, VT, USA), using Gen 5 software. 

In each well of the microplate, 150 µL of a fluorescein solution (40 nM) in phosphate buffer pH 7.4 was prepared. Then, 25 µL of the diluted extract at known concentrations were added to be incubated at 40 °C for 7 min in the microplate reader. After this time, 25 µL of APPH solution was added, resulting in a final volume of 200 µL in each plate. The blank was made using a phosphate-buffered solution at pH 7.4, instead of the sample, according to the methodology described by Pilaquinga et al. [46].

Fluorescence decay curves were normalized (F/F_0_), and the area under the curve (AUC) was calculated for both the samples and Trolox (Sigma-Aldrich, St. Louis, MO, USA), which was used as standard. After obtaining the Trolox calibration curve, the AUC of the samples was interpolated to finally express the results as µmol Trolox equivalent/g sample dry weight. Sample measurements were performed in triplicate for each extract.

### 3.9. Experimental Design

A central composite design was applied to optimize the total polyphenolic content and the antioxidant capacity of the walnut green husks extracts after UAE. This design allows the study of two factors: solid/solvent ratio (g/mL) and ethanol/water ratio (*v/v*) at three levels, generating 10 experimental runs. It considers central points that validate the design if the standard deviation between them is lower than 5% [47]. Independent of that, three replicates of the design were performed, and the mean data with the corresponding standard deviations are reported. Table 5 shows the variables and levels of the design. The low, medium, and high levels were defined, considering that the solvent volume increased from 1:10 to 1:30, and it was selected according to the preliminary studies. The response variables analyzed in the design were total polyphenol content (mg GAE/g sample dry weight) and antioxidant activity through three methods, DPPH (mg Trolox/g sample dry weight), FRAP (mg FeSO_4_/g sample dry weight), and ORAC (µmol Trolox/g sample dry weight). The experimental runs were randomized.

The response variables were maximized with a desirability function, i.e., the highest concentration of total polyphenols and the most significant antioxidant capacity. The desirability function method was applied, which helped to determine the combination of experimental factors that simultaneously optimized multiple responses. Model validation was performed with an additional set of three independent trials using the optimized design conditions.

### 3.10. Kinetics of Bacterial Growth

The kinetic growth of *Escherichia coli* ATCC 25,922 (Institute of Public Health, Chile) in the presence of extracts from walnut green husk was determined, following the methodology described by Celis-Cofré et al. [48]. A mixture (200 µL) containing: concentrated Mueller Hinton broth (Biokar Diagnostics, Beauvais, France), fresh bacteria at a concentration of 1 × 10^6^ UFC mL^−1^, and extracts from walnut green husk at different concentrations (16; 32; 48; 64; 80; 96 µg/mL) were added to a microplate (Bottger, Chicopee, MA, USA). Then, samples were incubated at 37 °C for 24 h on a microplate reader (Thermo Fisher, Multiskan Go, Vantaa, Finland), which recorded the absorbance of the sample at 625 nm every 1 h.

#### Kinetic Model

The modified Gompertz model (Equation (2)) was used to determine the effect of the concentration of the optimized extracts of the walnut green husks on the growth kinetics of a pathogenic bacteria, such as *Escherichia coli* [49].

(2) $$\ln\frac{D}{D_{0}}=A_{s}\exp\left\{-\exp\left[\frac{\mu_{\max}e}{A_{s}}\left(\lambda -t\right)+1\right]\right\},$$

where *D* is the microbial growth quantified in absorbance values at 625 nm (UA), *D*_0_ is the absorbance value at time zero (UA), *A_s_* is the asymptotic value of the maximum absorbance value (adimensional), *µ*_max_ is the maximum growth rate (h^−1^), *λ* is the lag phase (h), *t* the time (h), and *e* is the corresponding 2.718 number.

Additionally, the inhibition of *E. coli* was calculated according to Equation (3) [38]:

(3) $$I(\%)=\frac{\left(A_{c}-A_{s}\right)}{A_{c}}\times 100\%,$$

where *A_c_* is the asymptotic value of the maximum absorbance value of the control sample (*E. coli*) and *A_s_* is the asymptotic value of the maximum absorbance value of the extract (OH and CH) in the presence of the *E. coli*.

### 3.11. Statistical Analysis

All experiments were run in triplicate. Data are reported as the mean value with their corresponding standard deviation. ANOVA tests were performed to determine statistical differences in responses, i.e., the total phenolic content, antioxidant activity, and kinetic growth parameters, using Statgraphics Centurion XVI^®^ software (StatPoint Technologies Inc., The Plains, VA, USA).

## 4. Conclusions

The influence of the ripeness stage on the antioxidant and antimicrobial properties of the walnut green husk extract was demonstrated. The highest antioxidant and antimicrobial activities were obtained on the ripe sample with an open husk (OH), attributed to the different polyphenol profile related to the mechanical stress that occurs when the husk opens, compared to the unripe sample with a closed husk (CH). Moreover, walnut green husk extracts were able to inhibit the growth of Gram (−) bacteria such as *Escherichia coli*, proving the presence of polyphenols with antimicrobial capacity on the extracts.

The obtained results demonstrate the technological potential of the walnut green husk, a by-product of the walnut industry, as an economical source of antioxidant and antimicrobial agents for the food industry, considering the current circular economy trend. The elaboration of natural active additives based on the walnut green husk, principally the open husk (OH), could be used to replace synthetic additives currently used in food and beverages to prolong their shelf life.

## Figures and Tables

**Table 1:** Proximal analysis of the walnut green husks.

Percent (%)	Open Husk (OH)	Closed Husk (CH)
Moisture	11.716 ^a^ ± 0.147	14.074 ^b^ ± 0.679
Proteins	4.694 ^a^ ± 0.190	4.452 ^a^ ± 0.129
Lipids	1.385 ^a^ ± 0.788	1.610 ^a^ ± 0.129
Ashes	18.515 ^a^ ± 0.949	17.509 ^a^ ± 1.393
Crude fiber	44.700 ^a^ ± 0.018	44.800 ^a^ ± 0.027
Non-nitrogen extract	18.918 ^a^ ± 0.051	17.243 ^a^ ± 1.806

OH: ripe walnuts green husk (open husk); CH: unripe walnuts green husk (closed husk). * Different letters (a, b) indicate significant differences (*p* < 0.05) between samples.

**Table 2:** Polyphenolic compounds detected by HPLC from walnut green husks samples.

Polyphenol	Detection		Control Extraction		Optimized Extraction	
	DAD (nm)	FLD (nm)	OH	CH	OH	CH
Gallic acid	272	-	✓	✓	✓	✓
Protocatechuic acid	260–290	-	✓	✓	✓	✓
Catechin	278	280–318	✓	✓	-	✓
Caffeic acid	322	-	✓	-	-	-
Ferulic acid	322	332–445	✓	✓	✓	✓
Polydatin	306–318	320–395	✓	-	-	-
Hesperetin	284	-	-	-	-	-
Resveratrol	306–318	323–390	-	-	-	-
Quercetin	254–370	-	✓	✓	-	-
Myricetin	250–370	-	-	-	-	-
Kaempferol	270–366	-	✓	✓	-	-
Hesperidin	288	-	✓	✓	-	-
Juglone	235–275	-	-	-	✓	✓

OH: ripe walnuts green husk (open husk); CH: unripe walnuts green husk (closed husk); DAD: diode array detector; FLD: fluorescence detector.

**Table 3:** Experimental results for samples from the central composite design.

Independent Variables			Responses Variables			
Run	X_1_: Solid/Solvent Ratio (g/mL)	X_2_: Ethanol/Water Ratio (*v/v*)	Y_1_: TPC (mg GAE/g Sample dw)	Y_2_: DPPH (mg Trolox/g Sample dw)	Y_3_: FRAP (mg FeSO_4_/g Sample dw)	Y_4_: ORAC (µmol Trolox/g Sample dw)
1	1/20	50/50	105.99 ± 0.85	152.3 ± 3.0	102.4 ± 2.7	130 ± 1.4
2	1/30	75/25	102.21 ± 0.71	199.4 ± 2.8	123.7 ± 3.4	182.3 ± 2.5
3	1/10	25/75	31.17 ± 0.18	75.4 ± 1.1	65.2 ± 0.2	78.4 ± 0.86
4	1/10	75/25	45.95 ± 0.10	91.6 ± 0.6	83.1 ± 1.4	121.81 ± 0.64
5	1.4/40	50/50	67.02 ± 0.12	153.0 ± 0.8	78.4 ± 1.7	142.4 ± 4.4
6	1/30	25/75	57.07 ± 0.40	86.6 ± 1.9	59.6 ± 0.9	102.2 ± 0.86
7	1/20	50/50	106.01 ± 0.85	153.8 ± 1.5	96.6 ± 0.2	137.07 ± 0.06
8	1.4/14	50/50	50.12 ± 0.04	89.3 ± 0.1	80.5 ± 2.0	102.95 ± 0.38
9	1/20	35/65	65.05 ± 0.10	127.8 ± 0.5	81.2 ± 3.3	154.83 ± 1.08
10	1/20	65/35	82.43 ± 0.02	166.4 ± 0.9	120.3 ± 3.4	167.3 ± 0.84

TPC: total phenolic content; DPPH radical scavenging activity; FRAP: ferric reducing antioxidant power; ORAC: oxygen radical absorbance capacity; GAE: gallic acid equivalents; dw: dry weight. Mean data with their corresponding standard deviations are reported for each parameter.

**Table 4:** Comparison of the kinetic parameters of growth of *Escherichia coli* by adding extracts of the walnut green husks.

Extract (µg/mL)		Kinetic Parameters			*I* (%)	RMS (%)
		*A_s_* (adim)	*µ*_max_ (h^−1^)	*λ* (h)		
*Escherichia coli*		0.52 ^a^ ± 0.02	0.09 ^a^ ± 0.002	1.74 ^b^ ± 0.06	---	5.0
OH	16	0.26 ^b^ ± 0.05	0.03 ^b^ ± 0.01	1.57 ^b^ ± 0.14	44.4 ± 3.7	5.0
	32	0.25 ^bc^ ± 0.01	0.03 ^bc^ ± 0.004	1.25 ^b^ ± 0.21	51.6 ± 2.2	7.0
	48	0.22 ^bcd^ ± 0.05	0.013 ^efg^ ± 0.005	3.90 ^c^ ± 0.09	63.4 ± 2.0	12.0
	64	0.22 ^bcd^ ± 0.03	0.009 ^fgh^ ± 0.003	3.70 ^c^ ± 0.60	62.0 ± 0.1	9.8
	80	0.04 ^f^ ± 0.005	0.004 ^gh^ ± 0.001	5.00 ^d^ ± 0.04	78.4 ± 7.5	8.7
	96	0.04 ^g^ ± 0.005	0.003 ^h^ ± 0.001	3.35 ^c^ ± 0.57	93.2 ± 1.0	8.1
CH	16	0.26 ^b^ ± 0.02	0.03 ^bc^ ± 0.004	1.62 ^b^ ± 0.15	50.8 ± 3.2	5.3
	32	0.25 ^bc^ ± 0.01	0.024 ^cd^ ± 0.005	1.55 ^b^ ± 0.10	52.5 ± 1.6	6.2
	48	0.23 ^bcd^ ± 0.03	0.018 ^de^ ± 0.001	2.22 ^b^ ± 1.16	58.7 ± 2.0	7.4
	64	0.19 ^cde^ ± 0.01	0.016 ^ef^ ± 0.003	1.95 ^b^ ± 1.23	63.1 ± 2.6	9.4
	80	0.19 ^de^ ± 0.03	0.013 ^ef^ ± 0.004	1.92 ^b^ ± 0.05	66.1 ± 4.7	6.3
	96	0.15 ^ef^ ± 0.03	0.01 ^fg^ ± 0.001	0.00 ^a^ ± 0.00	68.9 ± 2.4	10.0

Different letters (a, b, c, d, e, f, g, h) indicate significant differences (*p* < 0.05) between samples. OH (open husk); CH (closed husk) samples. *A_s_*: asymptotic value of the maximum absorbance value (adimensional); *µ*_max_: the maximum growth rate (h^−1^); *λ*: lag phase (h), *I*: percentage inhibition of *E. coli* (%); and RMS: root mean square (%).

**Table 5:** Variables and levels of the central composite experimental design.

**Independent Variables**	**Level**		
	**Low (−1)**	**Medium (0)**	**High (1)**
X_1_: solid/solvent ratio (g/mL)	1:10	1:20	1:30
X_2_: ethanol/water ratio (*v/v*)	25/75	50/50	75/25
**Responses Variables**	**Goal**		
Y_1_: TPC (mg GAE/g sample dw)	Maximize		
Y_2_: DPPH (mg Trolox/g sample dw)			
Y_3_: FRAP (mg FeSO_4_/g sample dw)			
Y_4_: ORAC (µmol Trolox/g sample dw)			

TPC: total phenolic content; DPPH radical scavenging activity; FRAP: ferric reducing antioxidant power; ORAC: oxygen radical absorbance capacity; GAE: gallic acid equivalents; dw: dry weight.

## Data Availability

The data presented in this study are available on request from the corresponding author. The data are not publicly available due to it is part of Daniela Soto-Madrid Ph.D. thesis, which is not published yet.
